# A comprehensive analysis of BMI prediction using machine learning and biochemical markers: Insights from NHANES data

**DOI:** 10.1097/MD.0000000000042781

**Published:** 2025-07-11

**Authors:** Xiaoli Chen, Bingyu Zhu, Haiyang Jiang, Haiyan Lan, Chunyu Xie, Hongmei Lin, Yonglingzi Chen, Hong Chen

**Affiliations:** a Department of Gastrointestinal Surgery, Zhongjiang County People’s Hospital, Deyang, Sichuan, China; b Department of Urology I, The Third Affiliated Hospital of Kunming Medical University (Peking University Cancer Hospital Yunnan, Yunnan Cancer Hospital, Cancer Center of Yunnan Province), Kunming, Yunnan, China.

**Keywords:** body mass index, machine learning, obesity, predictive model, renal function

## Abstract

This study investigates the relationship between obesity and common biochemical markers, focusing on renal function indicators, using machine learning models to diagnose body mass index (BMI) and identify clinically relevant markers associated with it. Data from 4236 individuals in the 2017–2018 National Health and Nutrition Examination Survey dataset were used for model development, and 4748 individuals from the 2015–2016 National Health and Nutrition Examination Survey dataset were used for external validation. We explored the use of decision trees, balanced random forest (BRF), extreme gradient boosting, artificial neural networks, and logistic regression (LR) to distinguish overweight from normal weight. Feature selection was performed with a genetic algorithm, and the data were split into 80% training and 20% validation sets. Model performance was assessed using the area under the receiver operating characteristic curve (AUC), calibration plots, and learning curves, with key metrics including accuracy, precision, specificity, sensitivity, and F1 score. BRF and LR models showed reliable accuracy, generalizability, and stability. The BRF model achieved an AUC of 0.757 on the test set and 0.768 on the training set, while the LR model achieved an AUC of 0.779 on the test set and 0.783 on the training set. External validation showed similar results, with test set AUCs of 0.765 and 0.806 for BRF and LR, respectively. Calibration curves indicated good model calibration and learning curves suggested no significant overfitting. In conclusion, we developed accurate and generalizable machine learning models. Our findings highlight the influence of hepatic and renal function markers and socioeconomic factors on BMI, with low serum phosphorus levels notably associated with high BMI. These results enhance the understanding of serum phosphorus’s impact on obesity.

## 1. Introduction

Obesity has long been a serious public health concern.^[1,2]^ The World Health Organization classifies individuals with a body mass index (BMI) of 25 or higher as overweight and those with a BMI of 30 or higher as obese.^[3]^ According to the 2007 National Health and Nutrition Examination Survey (NHANES), 63% of Americans were classified as overweight and 26% as obese.^[4]^ These proportions have been increasing annually.^[5,6]^ World Health Organization describes overweight and obesity as abnormal or excessive fat accumulation that may impair health.^[7]^ Numerous studies have shown that individuals with a BMI over 25 are at increased risk for respiratory, cardiovascular diseases, acute kidney injury, and infections.^[7–10]^ While some research has focused on the relationship between BMI and factors such as bone density, blood glucose, and triglycerides,^[11,12]^ there is limited research on common biochemical markers related to obesity, particularly renal function indicators. Although some studies have noted the association of BMI with severe late-stage chronic kidney disease and nonalcoholic fatty liver disease,^[7]^ there is a lack of systematic research on specific markers, which is a gap in clinical research.

Machine learning (ML) has proven to be a promising tool in the medical field. Recent studies have demonstrated that these methods can develop effective diagnostic and predictive tools for identifying various diseases.^[13]^ ML, integrating techniques from statistics, computer science, and artificial intelligence, offers new solutions for uncovering complex relationships and patterns that traditional statistical methods might miss. These methods can be broadly applied to large-scale databases.^[14]^ There are existing studies employing ML to explore factors related to obesity.^[12,15,16]^ For example, Liu et al^[17]^ developed an ML model associated with BMI using gut microbiota. Lin et al^[18]^ also established an ML model using general population data and blood glucose levels. However, there is limited research on developing ML models using common biochemical markers, particularly liver and kidney function indicators, in relation to BMI. Only a few studies have used the Spearman test to analyze the relationship between blood markers and BMI,^[19]^ but the number of studies is limited. There is a research gap in the application of ML models to common biochemical markers, especially liver and kidney function indicators, and their association with BMI.

This study aims to use ML models and common biochemical markers from the NHANES database to develop diagnostic models distinguishing overweight and obese individuals from normal and underweight individuals based on BMI = 25. Additionally, this study seeks to identify important diagnostic factors distinguishing these groups through ML models. We collected data from the 2017–2018 and 2015–2016 NHANES populations for modeling and external validation, respectively, using logistic regression (LR), balanced random forest (BRF), extreme gradient boosting (XGBoost), adaptive boosting (AdaBoost), decision trees (DT), and artificial neural networks (ANN). We performed traditional statistical analyses by creating 2 sets of tables: one dividing the datasets at BMI = 25 into overweight and above (BMI ≥ 25) and normal and below (BMI < 25), and another dividing the datasets at BMI = 30 into obese and above (BMI ≥ 30) and overweight and below (BMI < 30) as supplementary parts of this study.

## 2. Materials and methods

### 2.1. Study design

This study employed a retrospective cohort design using data from the NHANES. NHANES is a critical program conducted by the National Center for Health Statistics (NCHS), collecting and analyzing data on the health and nutritional status of the US population since the 1960s. These data are utilized to assess the health and nutritional status of the American population, inform public health policies, and support health-related research.^[20]^

This cross-sectional survey was approved by the NCHS Ethics Review Board. The original protocol is available online at: https://www.cdc.gov/nchs/nhanes/about/erb.html. The features included in this study from the NHANES database were gender, age in years at screening, race/Hispanic origin, education level (adults 20+), marital status, annual household income, annual family income, alanine aminotransferase (ALT) (U/L), albumin (refrigerated serum, g/L), alkaline phosphatase (ALP) (IU/L), aspartate aminotransferase (U/L), bicarbonate (mmol/L), blood urea nitrogen (BUN) (mmol/L), chloride (mmol/L), creatine phosphokinase (CPK) (IU/L), creatinine (refrigerated serum, µmol/L), globulin (g/dL), glucose (refrigerated serum, mmol/L), gamma-glutamyl transferase (GGT) (IU/L), iron (refrigerated serum, µmol/L), lactate dehydrogenase (LDH) (IU/L), osmolality (mmol/kg), phosphorus (mmol/L), potassium (mmol/L), sodium (mmol/L), total bilirubin (µmol/L), total calcium (mmol/L), cholesterol (refrigerated serum, mmol/L), total protein (g/L), triglycerides (refrigerated serum, mmol/L), and uric acid (µmol/L). These features are available in the NHANES Demographics Data, Examination Data, and Laboratory Data. The outcome variable was defined based on BMI, with a cutoff of 25 to distinguish between normal/underweight and overweight/obese categories.

### 2.2. Feature selection rationale

The chosen features included demographic characteristics and common biochemical markers; gender, age at screening, race/Hispanic origin, education level, marital status, annual household income, and annual family income were selected as general demographic characteristics. These factors might influence BMI indirectly through metabolic and lifestyle differences. Biochemical markers such as ALT, albumin, ALP, aspartate aminotransferase, bicarbonate, BUN, chloride, CPK, creatinine, globulin, glucose, GGT, iron, LDH, osmolality, phosphorus, potassium, sodium, total bilirubin, total calcium, cholesterol, total protein, triglycerides, and uric acid were selected as they directly reflect metabolic activities within the body, including liver and kidney function, bone metabolism, fat metabolism, water–electrolyte balance, and tissue damage. These features were crucial for this study as they encompass a wide range of factors affecting BMI, thereby enhancing the predictive capability and accuracy of the model.

### 2.3. ML model strategy

To ensure the robustness and external validation feasibility of the model, data from the 2017–2018 and 2015–2016 cycles of the NHANES were utilized.

#### 2.3.1. Inclusion criteria

Retained 4236 individuals with complete biochemical markers, BMI, and socioeconomic background information (2017–2018 dataset) for model training and internal validation.

External validation used the 2015–016 NHANES dataset (4748 complete individual samples).

#### 2.3.2. Exclusion criteria

Removed samples with missing values, erroneous data, or outliers exceeding upper limits (as defined in the https://wwwn.cdc.gov/Nchs/Data/Nhanes/Public/2017/DataFiles/BIOPRO_J.htm) during data cleaning of the 2017–2018 dataset. No exclusion of outliers, upper-limit values, or erroneous data was applied to the 2015–2016 dataset to preserve its “real-world” characteristics for external validation. This approach aimed to more accurately simulate the model’s performance on new datasets, thereby enhancing the evaluation of its robustness and stability in real-world applications.

### 2.4. Sample classification and model construction

The dataset was divided into 80% for the training set (n = 3388) and 20% for the test set (n = 848). For the external validation dataset, the division was similarly set at 80% (n = 3798) for training and 20% (n = 950) for testing. In this study, the population with BMI ≥ 25 (primary data: n = 3148; external validation: n = 3463) was significantly larger than the population with BMI < 25 (primary data: n = 1088; external validation: n = 1285).

To address the class imbalance, we attempted to use Synthetic Minority Over-sampling Technique,^[21]^ Edited Nearest Neighbor undersampling,^[22,23]^ and a combination of both methods. However, we observed that models built using these balancing methods performed worse than models without balancing. Therefore, we did not apply these sampling methods during feature selection or model construction. Instead, we utilized the “class_weight” parameter adjustment to correct for the imbalance. As a result, the model was more reflective of real-world conditions.

We used a genetic algorithm (GA) for feature selection in each model. GA is a heuristic optimization algorithm inspired by biological evolution. It solves optimization problems through processes such as natural selection, genetics, and mutation. In GA, candidate solutions, called individuals, are typically represented using binary encoding. The algorithm generates an initial population of individuals randomly and evaluates their fitness to determine their survival and reproduction capabilities.^[24]^

Conventional feature selection methodologies, exemplified by Least Absolute Shrinkage and Selection Operator , exhibit inherent limitations in detecting complex nonlinear interdependencies within high-dimensional datasets due to their linear constraint frameworks. In contrast, GA demonstrates enhanced adaptability through systematic exploration of expanded solution spaces to identify globally optimal feature subsets. Although Least Absolute Shrinkage and Selection Operator was incorporated as a comparator, GA consistently exhibited superior performance in both selection efficiency and robustness metrics, thereby justifying its adoption as the principal methodology in this investigation.

### 2.5. Model development and selection

To comprehensively evaluate the performance and stability of different algorithms, 6 ML models were developed and compared, including LR, DT, BRF, XGBoost, AdaBoost, and ANN.

### 2.6. Model descriptions

- LR: A statistical model for binary classification. Despite its name, it is a classification algorithm using a logistic function (sigmoid function) to map the linear regression output to probabilities between 0 and 1 for classification.^[25]^
- DT: A nonparametric supervised learning method used for classification and regression tasks. It recursively splits the dataset into smaller subsets and creates simple decision rules at each split to form a tree structure. DTs are easy to interpret but prone to overfitting.^[26]^
- Ensemble learning methods: Ensemble learning is a method that enhances model performance by aggregating the predictions of multiple base learners (i.e., individual models). The underlying principle is to combine several weak learners in such a way that their collective performance achieves superior generalization compared to any single learner.^[27]^ These methods combine multiple base learners to improve model performance. They include Random Forest (RF), BRF, XGBoost, and AdaBoost. RF is an ensemble method based on DTs, combining the results of multiple DTs trained on bootstrap samples to enhance accuracy and robustness. BRF is an improved version of RF designed to handle class imbalance by treating each class more equally during training.^[28,29]^ XGBoost is an efficient gradient-boosting algorithm that builds new DTs iteratively to correct previous errors.^[30]^ AdaBoost constructs a strong classifier by combining multiple weak classifiers, typically small DTs, with weight adjustments to focus on misclassified samples.^[31]^
- ANN: A ML model simulating the structure and function of the human brain’s neural network. ANNs consist of input, hidden, and output layers, with each layer containing multiple neurons. Neurons transform input signals into output signals through weighted sums and activation functions. ANNs can handle complex nonlinear relationships and improve performance through backpropagation algorithms.^[32]^

### 2.7. Model parameters and internal validation

Parameter tuning primarily relied on 10-fold grid search cross-validation to determine hyperparameters. For LR, categorical variables were converted to dummy variables, and the first category was deleted to avoid multicollinearity, ensuring model stability.^[33]^ To ensure model reliability, we employed the bootstrap method, resampling 2000 times to internally validate model scores and area under the receiver operating characteristic curves (AUCs) on the test set. Additionally, we used bootstrap + out-of-bag (OOB) validation, specific to RF, to supplement bootstrap validation. Model stability was further assessed by adding noise and outliers. The noise was added by incorporating Gaussian noise with a standard deviation of 0.1 to each feature of the test set. Outliers were introduced by randomly selecting 10% of the test set samples and increasing each feature value by 5. To comprehensively assess model performance, a suite of established evaluation metrics was employed, encompassing accuracy (ACC), sensitivity, specificity, precision, F1-score, and the AUC.

### 2.8. Model parameterization strategy

In addition to the 10-fold cross-validation mentioned earlier, to prevent model overfitting, an early stopping strategy was incorporated to improve the performance of the learned function. To ensure model stability and reproducibility, the random seed was consistently set to 42. Details of the parameter tuning process are provided in Table S1–S6, Supplemental Digital Content, https://links.lww.com/MD/P159.

### 2.9. Statistical analysis

The characteristics and baseline information of different patient subsets are presented in Tables 1 and 2. Table 1 shows the characteristics of BMI < 25 and ≥ 25, while Table 2 shows the characteristics of BMI < 30 and ≥ 30. This was done to supplement our model development by studying the distinctions between overweight and mildly obese individuals, in addition to those between normal-weight and overweight individuals. We conducted the statistical analysis using Python version 3.10.6 (Python Software Foundation, Beaverton).

**Table 1:** Baseline data for patients with BMI ≥ 25 and < 25.

	Overweight and fat (n = 3148)	Normal (n = 1088)	*χ*^2^/*F*/*N*	df/Cliff’s Delta	*P* value
Gender			8.66	1	.0033
Male	1539	475			
Female	1609	613			
Age	54 (38~65)	49 (31~64)	5.419	0.11	<.001
Race			157.353	4	<.001
Mexican American	479	79			
Other Hispanic	307	65			
Non-Hispanic White	1177	396			
Non-Hispanic Black	704	212			
Other race, including multiracial	481	336			
Education level, adults 20+			34.120	4	<.001
Less than 9th grade	253	61			
9–11th grade (includes 12th grade with no diploma)	335	131			
High school graduate/GED or equivalent	761	263			
Some college or AA degree	1090	310			
College graduate or above	709	323			
Marital status			21.984	5	<.001
Married	1638	530			
Widowed	249	75			
Divorced	367	119			
Separated	112	36			
Never married	504	242			
Living with partner	278	86			
Annual household income			22.879	13	.043
$0–$ 4,999	79	33			
$5000–$9999	97	29			
$10,000–$14,999	150	63			
$15,000–$19,999	199	83			
$20,000–$24,999	205	67			
$25,000–$34,999	364	113			
$35,000–$44,999	379	94			
$45,000–$54,999	234	77			
$55,000–$64,999	203	62			
$65,000–$74,999	163	61			
$20,000 and Over	118	50			
Under $20,000	46	14			
$75,000–$99,999	324	102			
$100,000 and over	587	240			
Annual family income			21.948	13	.056
$0–$4999	97	39			
$5000–$9999	105	34			
$10,000–$14,999	170	75			
$15,000–$19,999	209	85			
$20,000–$24,999	216	79			
$25,000–$34,999	383	120			
$35,000–$44,999	378	90			
$45,000–$54,999	228	75			
$55,000–$64,999	189	59			
$65,000–$74,999	157	58			
$20,000 and Over	93	30			
Under $20,000	46	15			
$75,000–$99,999	315	102			
$100,000 and over	562	227			
Alanine aminotransferase (ALT) (U/L)	19 (14~27)	15 (12~21)	11.948	0.243	<.001
Albumin, refrigerated serum (g/dL)	40 (38~42)	41 (39~43)	−9.766	−0.198	<.001
Alkaline phosphatase (ALP) (IU/L)	77 (64~93)	70 (57~85)	8.696	0.177	<.001
Aspartate aminotransferase (AST) (U/L)	19 (16~24)	19 (16~23)	0.273	0.006	.785
Bicarbonate (mmol/L)	26 (24~27)	26 (24~28)	−5.251	−0.107	<.001
Blood urea nitrogen (mg/dL)	5 (3.930~6.430)	5 (3.930~6.070)	3.522	0.072	<.001
Chloride (mmol/L)	101 (99~103)	101 (99~103)	1.385	0.028	.166
Creatine phosphokinase (CPK) (IU/L)	116 (76~186.250)	104.50 (74~164.250)	3.608	0.073	<.001
Creatinine, refrigerated serum (µmol/L)	76.020 (63.650~90.170)	72.490 (61~85.750)	5.293	0.107	<.001
Globulin (g/dL)	3.100 (2.800~3.400)	3 (2.800~3.300)	5.994	0.122	<.001
Glucose, refrigerated serum (mmol/L)	5.270 (4.880~5.830)	5 (4.720~5.440)	11.612	0.236	<.001
Gamma-glutamyl transferase (GGT) (IU/L)	23 (16~34)	17 (12~25)	13.270	0.269	<.001
Iron, refrigerated serum (µmol/L)	14.500 (10.900~18.600)	15.900 (12.000~20.600)	−6.680	−0.136	<.001
Lactate dehydrogenase (LDH) (IU/L)	156 (139~177)	149 (132~171)	6.727	0.137	<.001
Osmolality (mmol/kg)	281 (277~285)	280 (277~284)	3.641	0.074	<.001
Phosphorus (mmol/L)	1.139 (1.033~1.259)	1.162 (1.066~1.259)	−4.941	−0.100	<.001
Potassium (mmol/L)	4.100 (3.800~4.300)	4 (3.800~4.300)	1.855	0.038	.064
Sodium (mmol/L)	140 (138~142)	140 (139~142)	−0.244	−0.005	.807
Total bilirubin (µmol/L)	6.840 (5.130~10.260)	6.840 (5.130~10.260)	−4.622	−0.094	<.001
Total calcium (mmol/L)	2.325 (2.250~2.375)	2.325 (2.275~2.375)	−2.478	−0.050	.013
Cholesterol, refrigerated serum (mmol/L)	4.810 (4.163~5.560)	4.681 (4.060~5.456)	2.649	0.054	.008
Total protein (g/L)	71 (68~74)	72 (69~74)	−1.736	−0.035	.083
Triglycerides, refrigerated serum (mmol/L)	1.423 (1.039~2.043)	1.027 (0.756~1.505)	17.086	0.347	<.001
Uric acid (µmol/L)	327.100 (273.600~392.600)	285.500 (237.900~345)	14.419	0.293	<.001

**Table 2:** Baseline data for patients with BMI ≥ 30 and < 30.

	Fat (n = 1799)	Overweight and normal (n = 2437)	*χ*^2^/*F*/*N*	df/Cliff’s Delta	*P* value
Gender			9.050	1	<.001
Male	807	1207			
Female	992	1230			
Age	52 (37~64)	53 (35~66)	−0.586	−0.011	.558
Race			155.237	4	<.001
Mexican American	282	276			
Other Hispanic	152	220			
Non-Hispanic White	702	871			
Non-Hispanic Black	465	451			
Other race, including multiracial	198	619			
Education level, adults 20+			55.424	4	<.001
Less than 9th grade	123	191			
9–11th grade (Includes 12th grade with no diploma)	205	261			
High school graduate/GED or equivalent	458	566			
Some college or AA degree	669	731			
College graduate or above	344	688			
Marital status			8.716	5	.121
Married	881	1287			
Widowed	150	174			
Divorced	219	267			
Separated	71	77			
Never married	316	430			
Living with partner	162	202			
Annual household income			35.820	13	<.001
$0–$4999	47	65			
$5000–$9999	60	66			
$10,000–$14,999	88	125			
$15,000–$19,999	111	171			
$20,000–$24,999	117	155			
$25,000–$34,999	211	266			
$35,000–$44,999	223	250			
$45,000–$54,999	143	168			
$55,000–$64,999	134	131			
$65,000–$74,999	103	121			
$20,000 and Over	68	100			
Under $20,000	28	32			
$75,000–$99,999	174	252			
$100,000 and over	292	535			
			29.826	13	<.001
Annual family income					
$0–$4999	54	82			
$5000–$9999	64	75			
$10,000–$14,999	103	142			
$15,000–$19,999	121	173			
$20,000–$24,999	124	171			
$25,000–$34,999	222	281			
$35,000–$44,999	219	249			
$45,000–$54,999	136	167			
$55,000–$64,999	124	124			
$65,000–$74,999	99	116			
$20,000 and Over	54	69			
Under $20,000	27	34			
$75,000–$99,999	173	244			
$100,000 and over	279	510			
Alanine aminotransferase (ALT) (U/L)	19 (14~29)	17 (12~24)	9.780	0.176	<.001
Albumin, refrigerated serum (g/dL)	40 (38~42)	41 (39~43)	−15.073	−0.271	<.001
Alkaline phosphatase (ALP) (IU/L)	79 (65~95)	72 (60~88)	8.944	0.161	<.001
Aspartate aminotransferase (AST) (U/L)	19 (16~24)	19 (16~23)	−1.433	−0.026	.152
Bicarbonate (mmol/L)	25 (24~27)	26 (24~28)	−6.557	−0.118	<.001
Blood urea nitrogen (mg/dL)	5 (3.930~6.430)	5 (3.930~6.430)	0.979	0.018	.328
Chloride (mmol/L)	101 (99~103)	101 (99~103)	1.363	0.024	.173
Creatine phosphokinase (CPK) (IU/L)	116 (75~188)	109 (75~173)	2.243	0.040	.025
Creatinine, refrigerated serum (µmol/L)	75.140 (62.760~90.170)	75.140 (61.880~88.400)	1.225	0.022	.221
Globulin (g/dL)	3.100 (2.800~3.400)	3 (2.800~3.300)	8.379	0.150	<.001
Glucose, refrigerated serum (mmol/L)	5.330 (4.940~6.050)	5.110 (4.770~5.550)	10.398	0.187	<.001
Gamma-glutamyl transferase (GGT) (IU/L)	24 (17~36)	19 (13~29)	10.872	0.195	<.001
Iron, refrigerated serum (µmol/L)	14 (10.400~17.600)	15.800 (12~20.100)	−10.648	−0.191	<.001
Lactate dehydrogenase (LDH) (IU/L)	158 (141~180)	152 (135~172)	7.069	0.127	<.001
Osmolality (mmol/Kg)	281 (277~285)	281 (277~284)	2.706	0.049	<.001
Phosphorus (mmol/L)	1.130 (1.001~1.259)	1.162 (1.033~1.259)	−3.410	−0.061	<.001
Potassium (mmol/L)	4.100 (3.800~4.300)	4.100 (3.800~4.300)	1.113	0.020	.265
Sodium (mmol/L)	140 (138~142)	140 (139~142)	−0.771	−0.014	.441
Total bilirubin (µmol/L)	6.840 (5.130~8.550)	6.840 (5.130~10.260)	−7.176	−0.129	<.001
Total calcium (mmol/L)	2.300 (2.250~2.375)	2.325 (2.275~2.375)	−4.754	−0.085	<.001
Cholesterol, refrigerated serum (mmol/L)	4.784 (4.138~5.482)	4.784 (4.138~5.534)	−0.248	−0.004	.804
Total protein (g/L)	71 (68~74)	72 (69~74)	−3.425	−0.061	<.001
Triglycerides, refrigerated serum (mmol/L)	1.479 (1.084~2.134)	1.197 (0.847~1.739)	13.023	0.234	<.001
Uric acid (µmol/L)	339 (279.600~398.500)	303.300 (249.800~362.800)	12.109	0.217	<.001

Given the large sample size, we employed the Kolmogorov–Smirnov test to assess the normality of continuous variables.^[34]^ The homogeneity of variance between groups was tested using the Bartlett test.^[35]^ For normally distributed data with homogenous variances, we used independent samples *t* tests,^[36]^ while the Welch *t* test was applied to normally distributed data with heterogeneous variances.^[37]^ These results were described using mean ± standard deviation. For nonnormally distributed data, we used the Wilcoxon rank-sum test^[38]^ and described the data using median (25th–75th percentile) (M [P25–P75]). Categorical and ordinal data were presented as frequencies (%), and differences between groups were assessed using chi-squared tests for binary variables.^[39]^

The significance level was set at *α* = .05. Differences were considered statistically significant if *P* < .05.

By using these comprehensive statistical methods, we ensured robust analysis and interpretation of the data, enhancing the validity and reliability of our findings.

## 3. Statistical results and model performance

### 3.1. Statistical analysis results

The statistical analysis revealed significant relationships between specific demographic and biochemical markers and BMI categories. Specifically:

- Overweight versus obesity (BMI ≥ 25 vs BMI < 25): Gender, age, race, education level, marital status, annual household income, ALT, serum albumin, ALP, bicarbonate, BUN, CPK, serum creatinine, globulin, serum glucose, GGT, serum iron, LDH, osmolality, phosphorus, total bilirubin, total calcium, serum cholesterol, serum triglycerides, and uric acid were statistically significant.
- Normal/underweight versus overweight/obese (BMI ≥ 30 vs BMI < 30): Gender, race, education level, annual household income, annual family income, ALT, serum albumin, ALP, bicarbonate, CPK, globulin, serum glucose, GGT, serum iron, LDH, osmolality, phosphorus, total bilirubin, total calcium, total protein total, serum triglycerides, and uric acid showed significant differences.

### 3.2. Model performance

Our analysis identified BRF and LR as the top-performing models, demonstrating exceptional generalizability across multiple validation paradigms. Both algorithms maintained consistent accuracy scores above 0.700 in both training and test sets, with BRF showing remarkable stability in external validation (test AUC: 0.765 vs training AUC: 0.789) and LR achieving the highest discriminative performance in external testing (test AUC: 0.806 vs training AUC: 0.803), indicating superior out-of-distribution generalization capabilities.

Key findings from internal validation revealed statistically significant advantages for these models. BRF’s test set AUC confidence intervals (bootstrap: 0.744–0.766; bootstrap + OOB: 0.706–0.763) and LR’s corresponding intervals (bootstrap: 0.759–0.783; bootstrap + OOB: 0.737–0.787) substantially outperformed other models in precision and reliability. Both models demonstrated notable robustness to data perturbations, with BRF maintaining 0.709 accuracy under noise and 0.706 with outliers, while LR achieved 0.708 accuracy with noise and 0.719 with outliers—surpassing even complex models like XGBoost (noise ACC: 0.696) instability metrics.

**Figure 1.**
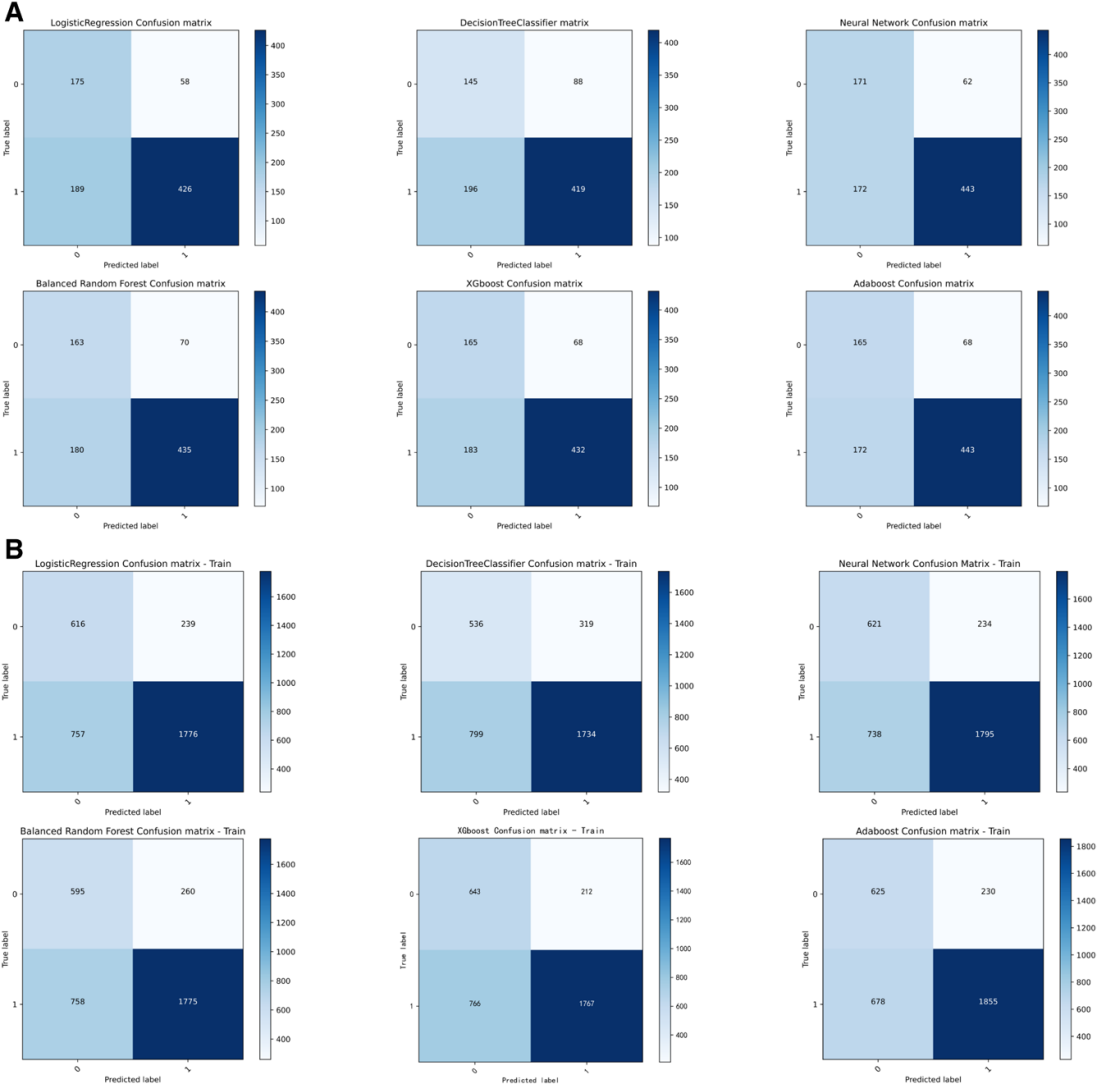
(A) Confusion matrix for model test set. (B) Confusion matrix for model train set.

**Figure 2.**
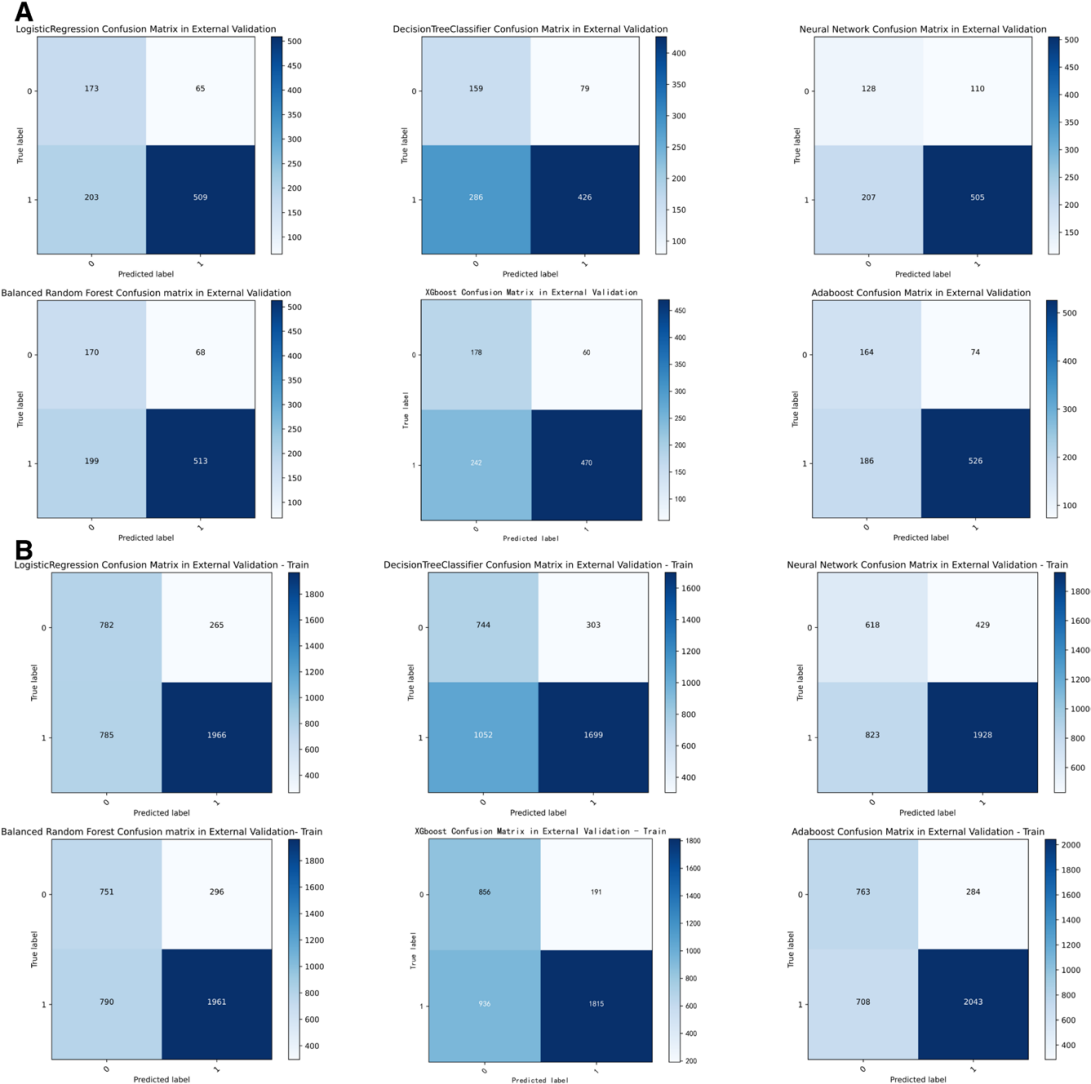
(A) Confusion matrix for external validation test set. (B) Confusion matrix for external validation train set.

**Figure 3.**
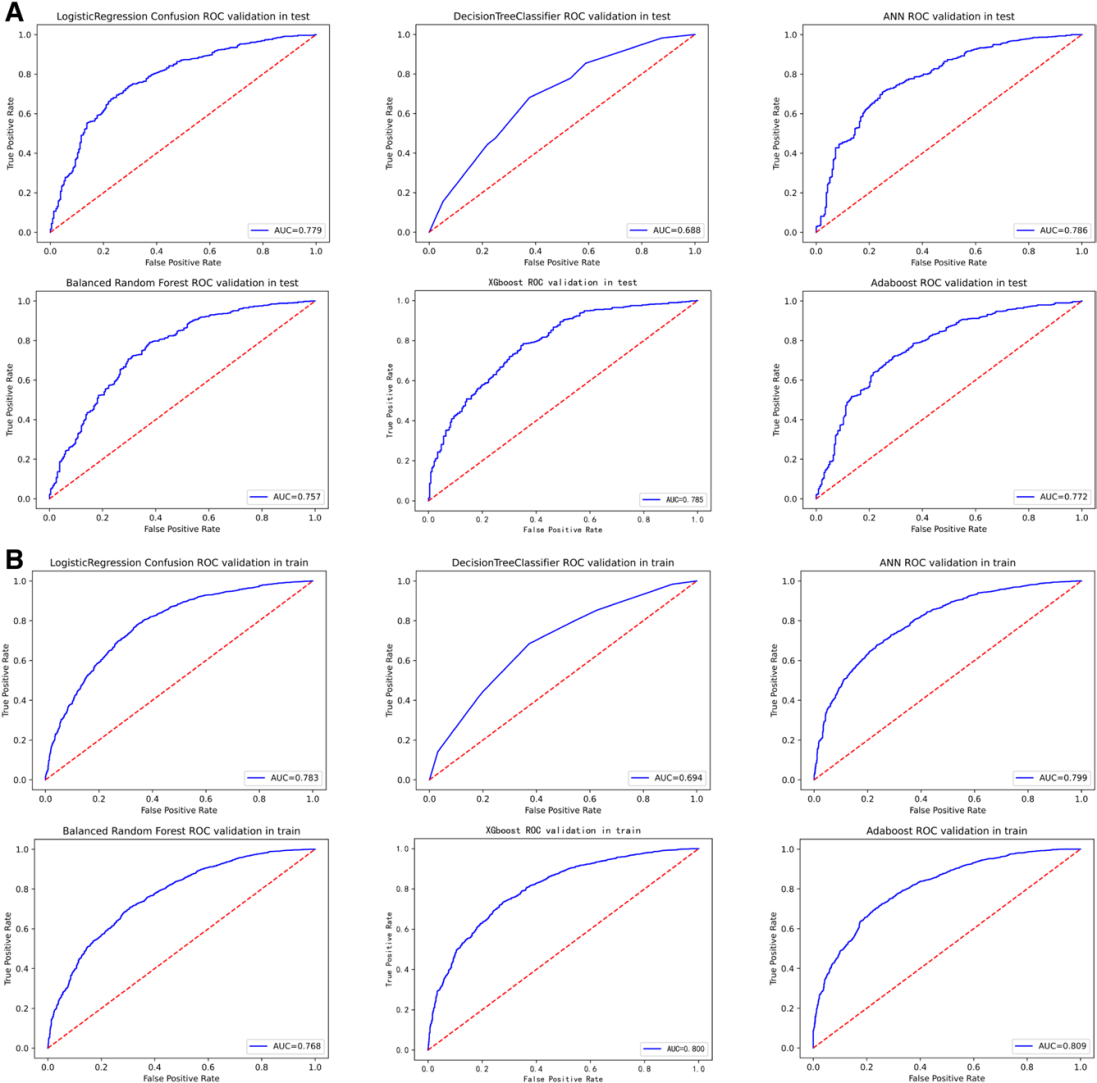
(A) ROC curve for model test set. (B) ROC curve for model train set. ROC = receiver operating characteristic.

**Figure 4.**
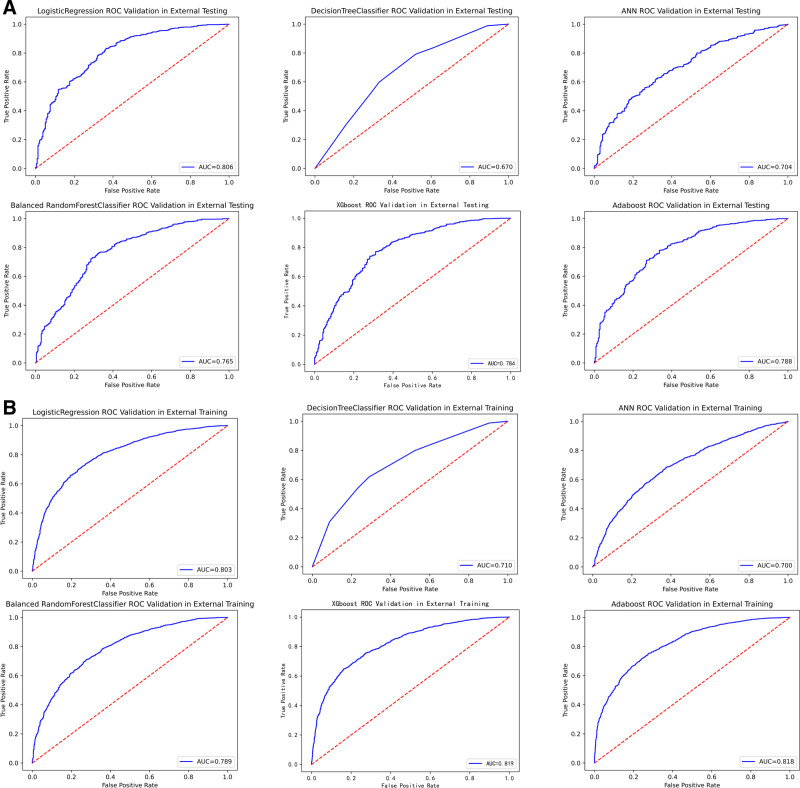
(A) ROC curve for external validation test set. (B) ROC curve for external validation train set. ROC = receiver operating characteristic.

**Figure 5.**
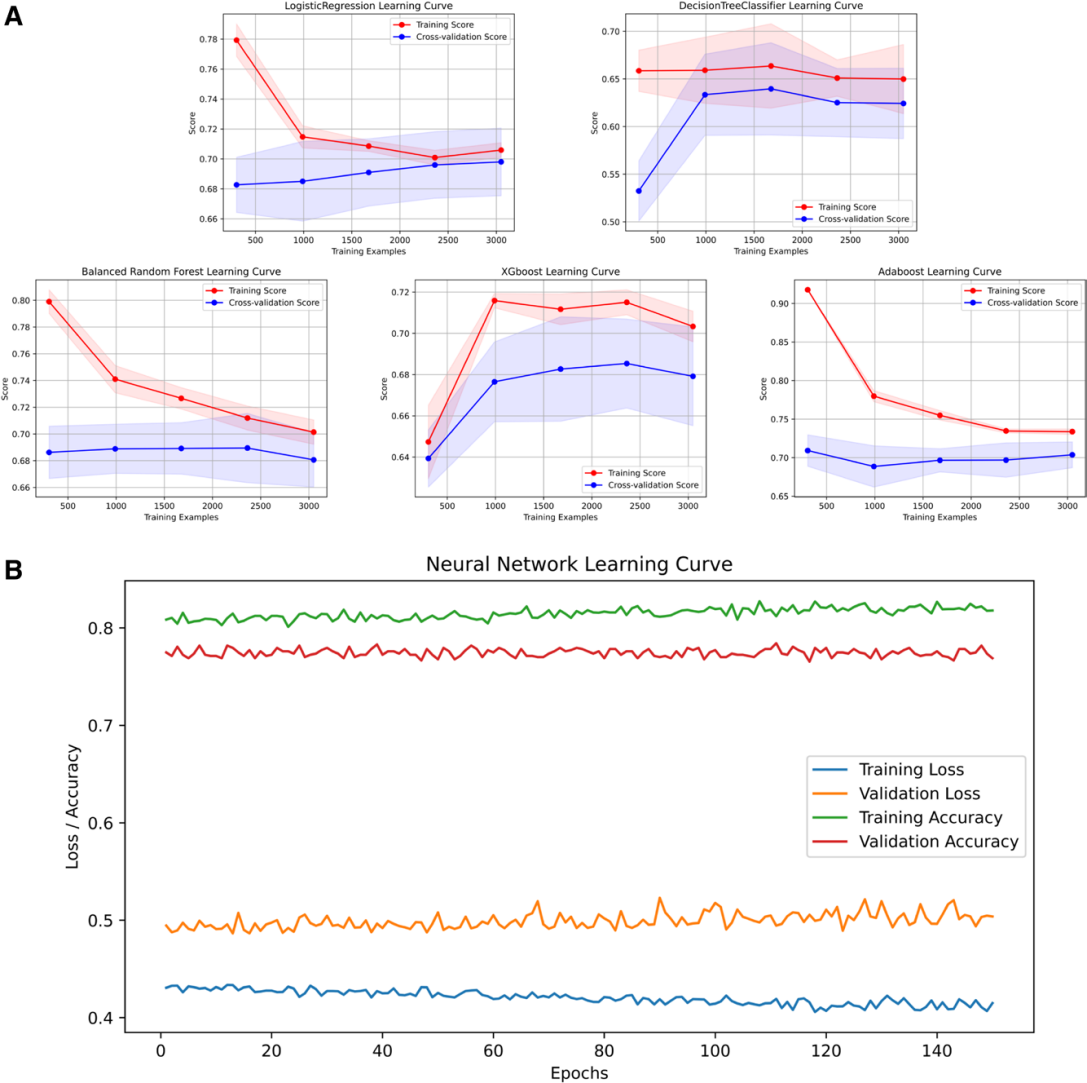
(A) Learning curve for the models. (B) Learning curve for ANN. ANN = artificial neural networks.

**Figure 6.**
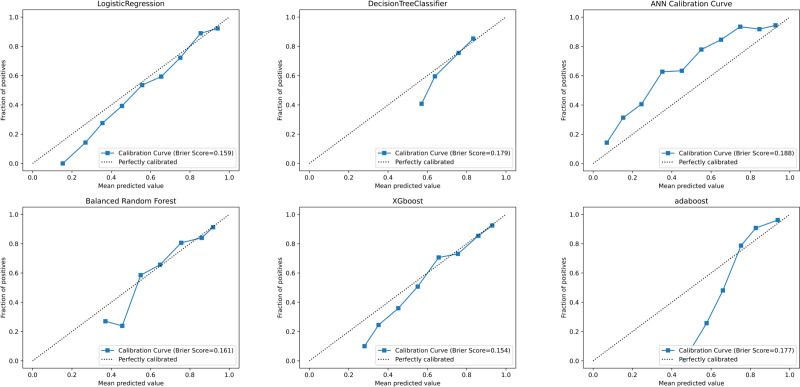
Calibration curve for the model.

While alternative approaches showed intermittent strengths—such as AdaBoost training accuracy of 0.732 and XGBoost test AUC of 0.785—their external validation performance (AdaBoost test AUC: 0.788; XGBoost test AUC: 0.784) consistently trailed LR’s benchmark. Simpler models like DT exhibited significant performance degradation (test ACC: 0.665, AUC: 0.688), further emphasizing BRF and LR’s optimal balance of model complexity and predictive reliability. The combination of high AUC preservation (≤3.5% drop from training to external test sets) and perturbation resistance positions these 2 models as the most clinically plausible choices for real-world deployment. Complete performance visualizations are available in Tables 3–6, as well as in Figures 1A, B, 2A, B, 3A, B, and 4A, B. Additionally, the learning curves and calibration curves, which show the average scores and standard deviations for the training and test sets, are provided in Figures 5A, B and 6.

**Table 3:** Confusion matrix scores for model test set.

	Accuracy rate (ACC)	Sensitivity (SEN) or regression rate (recall)	Specificity (SPE)	Precision (PRE)	F1-score	The area under the ROC curve (AUC)
DT	0.665	0.681	0.622	0.826	0.747	0.688
XGBoost	0.704	0.702	0.708	0.864	0.775	0.785
BRF	0.705	0.707	0.700	0.861	0.777	0.757
ANN	0.724	0.720	0.734	0.877	0.791	0.786
LR	0.709	0.693	0.751	0.880	0.775	0.779
AdaBoost	0.717	0.720	0.708	0.867	0.787	0.772

AdaBoost = adaptive boosting, ANN = artificial neural networks, BRF = balanced random forests, DT = decision trees, LR = logistic regression, XGBoost = extreme gradient boosting.

**Table 4:** Confusion matrix scores for model training set.

	Accuracy rate (ACC)	Sensitivity (SEN) or regression rate (recall)	Specificity (SPE)	Precision (PRE)	F1 score	The area under the ROC curve (AUC)
DT	0.670	0.685	0.627	0.845	0.756	0.694
XGBoost	0.711	0.698	0.752	0.893	0.783	0.800
BRF	0.700	0.701	0.696	0.872	0.777	0.768
ANN	0.713	0.709	0.726	0.885	0.787	0.799
LR	0.706	0.701	0.720	0.881	0.781	0.783
AdaBoost	0.732	0.732	0.731	0.890	0.803	0.809

AdaBoost = adaptive boosting, ANN = artificial neural networks, BRF = balanced random forests, DT = decision trees, LR = logistic regression, XGBoost = extreme gradient boosting.

**Table 5:** Confusion matrix: external validation (test).

	Accuracy rate (ACC)	Sensitivity (SEN) or regression rate (recall)	Specificity (SPE)	Precision (PRE)	F1 score	The area under the ROC curve (AUC)
DT	0.616	0.598	0.668	0.844	0.700	0.670
XGBoost	0.682	0.660	0.748	0.887	0.757	0.784
BRF	0.719	0.721	0.714	0.883	0.794	0.765
ANN	0.666	0.709	0.538	0.821	0.761	0.704
LR	0.718	0.715	0.727	0.887	0.792	0.806
AdaBoost	0.726	0.739	0.689	0.877	0.802	0.788

AdaBoost = adaptive boosting, ANN = artificial neural networks, BRF = balanced random forests, DT = decision trees, LR = logistic regression, XGBoost = extreme gradient boosting.

**Table 6:** Confusion matrix: external validation (train).

	Accuracy rate (ACC)	Sensitivity (SEN) or regression rate (recall)	Specificity (SPE)	Precision (PRE)	F1 score	The area under the ROC curve (AUC)
DT	0.643	0.618	0.711	0.849	0.715	0.710
XGBoost	0.703	0.660	0.818	0.905	0.763	0.819
BRF	0.714	0.713	0.717	0.869	0.783	0.789
ANN	0.670	0.701	0.590	0.818	0.755	0.700
LR	0.724	0.715	0.747	0.881	0.789	0.803
AdaBoost	0.739	0.743	0.729	0.878	0.805	0.818

AdaBoost = adaptive boosting, ANN = artificial neural networks, BRF = balanced random forests, DT = decision trees, LR = logistic regression, XGBoost = extreme gradient boosting.

### 3.3. AUC and model calibration curve

#### 3.3.1. AUC curve

AUC is an important metric for evaluating the performance of binary classification models, as it measures the model’s ability to distinguish between positive and negative classes. The vertical axis of the receiver operating characteristic curve represents the true positive rate (sensitivity), while the horizontal axis represents the false positive rate (1 − specificity). A higher AUC value indicates better model performance and a stronger discriminative ability.^[40]^

According to the AUC values reported in our study, the ANN model had the highest AUC value (0.786), indicating that it performed best in distinguishing positive and negative cases. All of the models we developed, except for the DT, achieved AUC values above 0.75, demonstrating strong classification capability.

#### 3.3.2. Model calibration curve

Calibration curves evaluate the reliability of probabilistic predictions by comparing predicted probabilities against observed event frequencies. The *x*-axis represents the mean predicted probability (binned into deciles), while the *y*-axis denotes the empirically observed probability. A perfectly calibrated model aligns with the 45° reference line, where predicted probabilities equal actual outcomes. Deviations from this diagonal indicate systematic prediction biases, such as consistent under- or overestimation of event risks.

The Brier score serves as a synthetic metric that quantifies both the calibration and discrimination capabilities of probabilistic models by calculating the mean squared error between predicted probabilities and observed outcomes. It achieves a theoretical minimum of 0 (perfect prediction accuracy) and a maximum of 1 (complete inaccuracy), with lower scores indicating superior performance. Based on clinical prediction model standards, Brier scores are interpreted as follows: ≤0.1: excellent calibration and discrimination; 0.1 to 0.25: clinically acceptable performance; >0.25: substantial miscalibration requiring algorithmic optimization.

In this study, we used the calibration curve and Brier score to evaluate the predicted probabilities of the model. The Brier scores for our models ranged from 0.1 to 0.2, indicating good calibration performance. Additionally, the deviation of the blue line in the calibration curve helped identify the direction of the model’s bias. If the curve is above the 45° diagonal, it indicates that the model is underpredicting probabilities, whereas if the curve is below the 45° diagonal, it indicates that the model is overpredicting probabilities. These results suggest that our model is reliable and stable when predicting the probability of BMI classification.

### 3.4. Model performance and generalization

Our results revealed that ANN and AdaBoost may exhibit signs of overfitting during model development. Additionally, the performance of XGBoost declined in external validation, indicating potential generalization issues. In contrast, the BRF and LR models demonstrated strong performance across several aspects, including model scores, internal validation, external validation, and robustness. Their superior generalization ability suggests that these models are well-suited for practical applications.

### 3.5. Model interpretation

#### 3.5.1. FI, SHAP, and LIME

**Figure 7.**
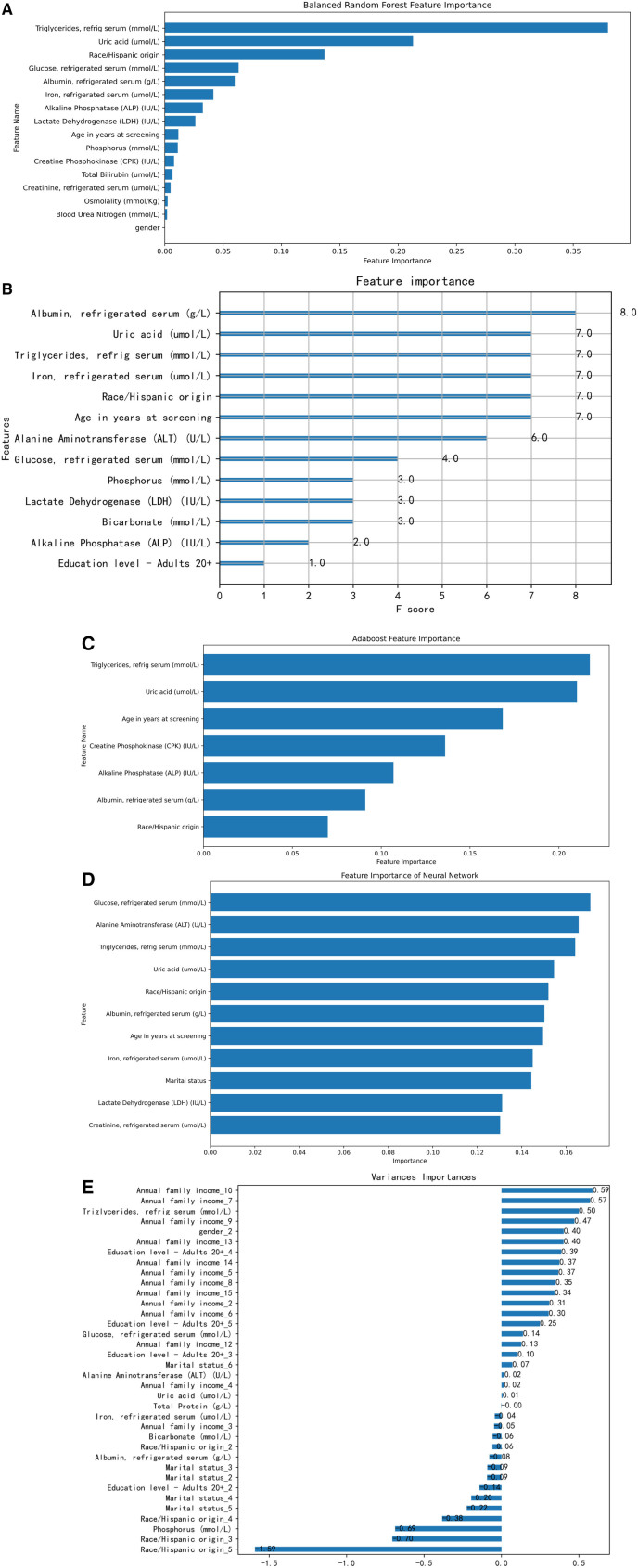
(A) BRF feature importance selection. (B) XGBoost feature importance selection. (C) AdaBoost feature importance selection. (D) ANN feature importance selection. (E) LR feature importance selection. AdaBoost = adaptive boosting, ANN = artificial neural networks, BRF = balanced random forests, LR = logistic regression, XGBoost = extreme gradient boosting.

**Figure 8.**
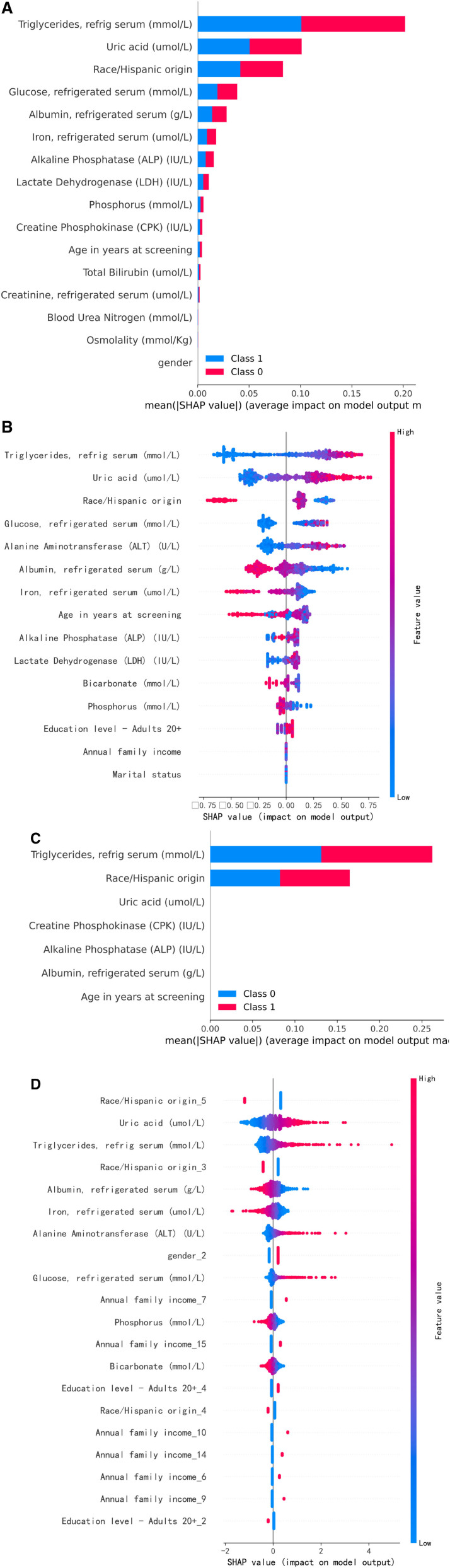
(A) SHAP for BRF. (B) SHAP for XGBoost. (C) SHAP for base_estimator. (D) SHAP for LR. BRF = balanced random forests, LR = logistic regression, SHAP = SHapley Additive exPlanations, XGBoost = extreme gradient boosting.

**Figure 9.**
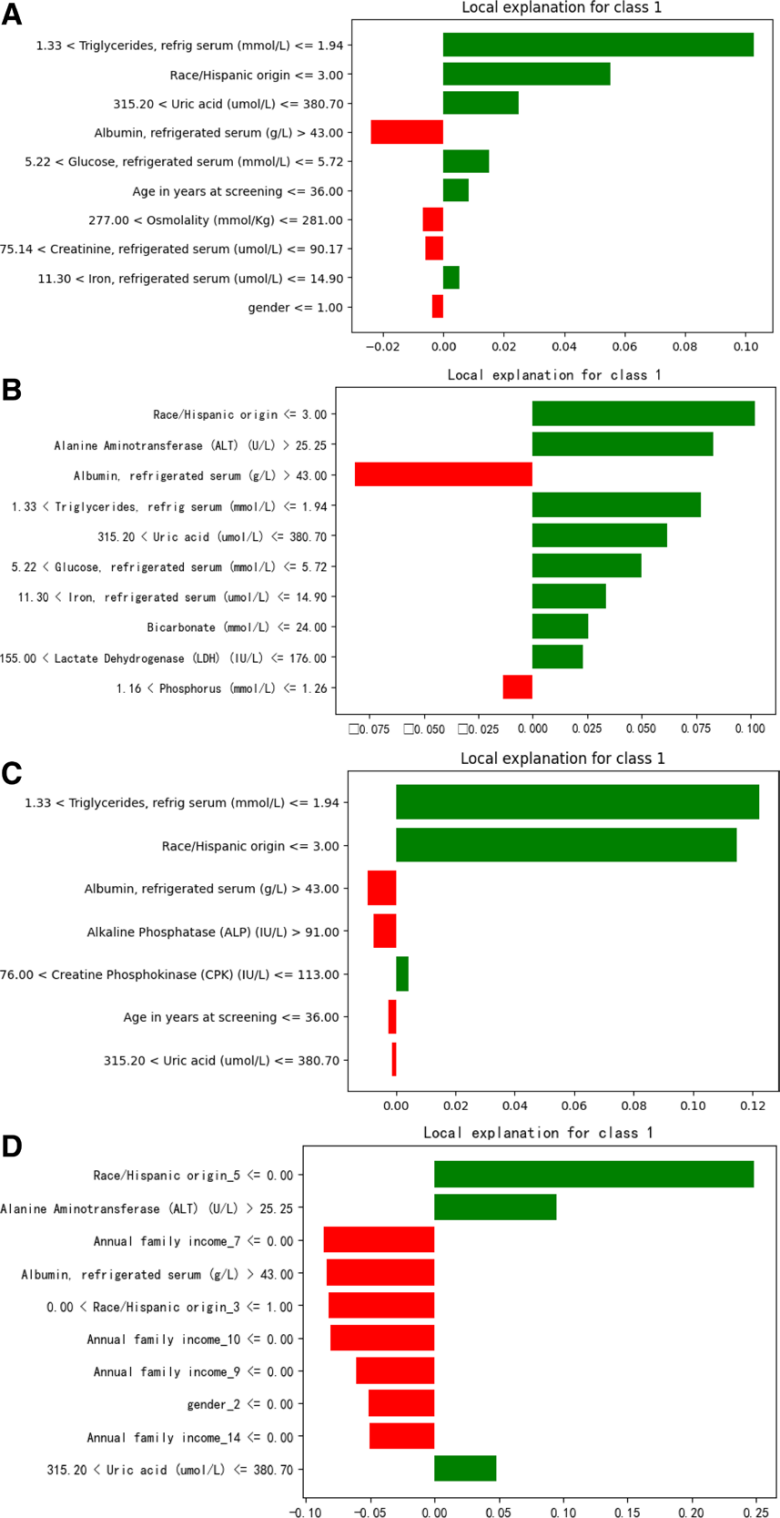
(A) LIME for BRF. (B) LIME for XGBoost. (C) LIME for base_estimator. (D) LIME for LR. BRF = balanced random forests, LR = logistic regression, LIME = local interpretable model-agnostic explanations, XGBoost = extreme gradient boosting.

We constructed Feature Importance (FI), SHapley Additive exPlanations (SHAP), and Local Interpretable Model-agnostic Explanations (LIME) visualizations for the BRF, XGBoost, AdaBoost, and LR models. Due to the complexity of the data and technical limitations, only FI was created for the ANN. Figures 7A–E, 8A–D, and 9A–D display the FI, SHAP, and LIME visualizations for the aforementioned models.

FI evaluates the impact of each feature on the model’s output. By measuring each feature’s contribution to model performance (e.g., accuracy or error), FI identifies the most critical features in model decision-making.^[41]^ SHAP is a game theory-based interpretation method that uses Shapley values to assign FI, ensuring consistent and fair explanations. SHAP values are derived from cooperative game theory, which allocates cooperative benefits. SHAP not only explains individual predictions but also provides global interpretations, offering a better understanding of the model’s behavior under different circumstances.^[42]^ LIME is a model-agnostic interpretation method that generates neighborhood data points in the input space and fits a simple, interpretable model (e.g., linear model) to approximate the complex model’s local behavior.^[42]^

In summary, LIME can explain single prediction instances of various ML models, including deep learning models. SHAP, on the other hand, is better suited for providing global FI evaluations and explanations. FI quickly shows the overall feature contributions of a model. Note that AdaBoost does not directly support LIME and SHAP; thus, we illustrate it using base learners.

### 3.6. FI in models

- XGBoost: Albumin, uric acid, and triglycerides are the most important features.
- BRF: Triglycerides, uric acid, race, and glucose are the most important features.
- AdaBoost: Triglycerides, uric acid, and age are the most important features.
- ANN: Glucose, ALT, triglycerides, and uric acid are the most important features.
- LR: Uses weight coefficients to determine FI. For example, the categorical variable “race” is decomposed into 2, 3, 4, and 5, with race_1 as the constant item. Race_1–5 correspond to Mexican American, other Hispanic, Non-Hispanic White, Non-Hispanic Black, and other race—including multiracial. In LR FI, annual family income and education level are positively correlated, while race and marital status are negatively correlated with the model.

### 3.7. SHAP analysis

- BRF and XGBoost: Triglycerides, uric acid, and race are the most important features.
- AdaBoost: Triglycerides and race are the most important features in the base learners.
- LR: Other race—including multiracial, uric acid, and triglycerides are the most important features.

### 3.8. LIME analysis

- BRF: Triglycerides, race, and uric acid are the most important features.
- XGBoost: Race, ALT, albumin, and triglycerides are the most important features.
- AdaBoost: Triglycerides, race, and albumin are the most important features in the base learners.
- LR: Other race—including multiracial, ALT, and annual family income are the most important features.

### 3.9. LR detailed analysis

LR model quantified the influence of biomarkers and demographic factors on the target variable through coefficients and statistical significance metrics, as detailed in Tables 7 and 8. Table 7, generated using statsmodels, reports coefficients, standard errors (precision of estimates), *z* values (significance ratios), *P* values (threshold: *P* < .05), and 95% confidence intervals, identifying significant predictors such as bicarbonate, serum phosphorus, female gender, and specific annual family income tiers. Table 8 integrates scikit-learn-derived feature impact magnitudes with statsmodels’ statistical rigor, expanding the list of significant variables to include glucose, uric acid, racial subgroups, and additional income segments.

**Table 7:** LR analysis: biomarkers and demographics.

Variable	Coefficient	Standard error	*z*	*P* > *z*	[0.025	0.975]
Constant	3.796770856	1.084002696	3.50254743	0.000460832	1.672164613	5.921377099
Alanine aminotransferase (ALT) (U/L)	0.022122523	0.004137902	5.346314283	8.97633E−08	0.014012384	0.030232661
Albumin, refrigerated serum (g/L)	−0.088944789	0.016652119	−5.341349629	9.22571E−08	−0.121582342	−0.056307236
Bicarbonate (mmol/L)	−0.055390053	0.018546301	−2.98658231	0.002821149	−0.091740134	−0.019039972
Glucose, refrigerated serum (mmol/L)	0.154820509	0.036546365	4.236276568	2.27257E−05	0.08319095	0.226450068
Iron, refrigerated serum (µmol/L)	−0.047228203	0.00740374	−6.378965946	1.78288E−10	−0.061739266	−0.03271714
Phosphorus (mmol/L)	−0.960968255	0.284582524	−3.376764818	0.000733437	−1.518739752	−0.403196757
Total protein (g/L)	−0.003198323	0.012360597	−0.258751493	0.795826986	−0.027424647	0.021028002
Triglycerides, refrigerated serum (mmol/L)	0.541727684	0.066828871	8.106192379	5.22307E−16	0.410745504	0.672709864
Uric acid (µmol/L)	0.007383215	0.000666623	11.07555156	1.64862E−28	0.006076659	0.008689772
Gender_2	0.383805891	0.108296798	3.544018811	0.000394077	0.171548066	0.596063715
Race/Hispanic origin_2	−0.000567051	0.22355183	−0.002536551	0.997976127	−0.438720586	0.437586485
Race/Hispanic origin_3	−0.680998233	0.167715448	−4.060438324	4.89807E−05	−1.009714471	−0.352281995
Race/Hispanic origin_4	−0.332476	0.182649438	−1.820295777	0.068713974	−0.690462319	0.02551032
Race/Hispanic origin_5	−1.569067373	0.177674602	−8.831129216	1.03624E−18	−1.917303194	−1.220831551
Education level—adults 20+_2	−0.202753833	0.227279212	−0.892091412	0.372343938	−0.648212903	0.242705237
Education level—adults 20+_3	0.025922066	0.213000207	0.121699724	0.903136832	−0.391550669	0.443394802
Education level—adults 20+_4	0.316550974	0.211157441	1.499122986	0.13384173	−0.097310006	0.730411954
Education level-Adults 20+_5	0.141347515	0.221145764	0.639159945	0.522718886	−0.292090219	0.574785248
Marital status_2	−0.040113094	0.182363122	−0.219962749	0.825900166	−0.397538245	0.317312057
Marital status_3	−0.007005433	0.156290173	−0.044823247	0.964248196	−0.313328542	0.299317676
Marital status_4	−0.202779476	0.247365081	−0.819757886	0.412354143	−0.687606126	0.282047174
Marital status_5	−0.189159416	0.12670931	−1.492861231	0.135473515	−0.4375051	0.059186268
Marital status_6	0.120224817	0.170915797	0.703415479	0.481796863	−0.21476399	0.455213624
Annual family income_2	0.379146059	0.344758181	1.09974492	0.271443277	−0.29656756	1.054859678
Annual family income_3	0.056390842	0.293459875	0.192158612	0.847617964	−0.518779944	0.631561629
Annual family income_4	0.097772811	0.284348081	0.343849026	0.730959828	−0.459539187	0.655084808
Annual family income_5	0.431755325	0.290787767	1.484778157	0.1376026	−0.138178226	1.001688876
Annual family income_6	0.392104339	0.268711812	1.459200233	0.144510005	−0.134561135	0.918769813
Annual family income_7	0.728088951	0.276918457	2.629253963	0.008557243	0.185338748	1.270839153
Annual family income_8	0.466295358	0.289963692	1.608116365	0.107809696	−0.102023035	1.034613751
Annual family income_9	0.596680034	0.302576436	1.971997694	0.04860987	0.003641117	1.189718952
Annual family income_10	0.756046756	0.319488099	2.366431674	0.017960489	0.129861589	1.382231923
Annual family income_12	0.369307577	0.352529428	1.047593612	0.294825882	−0.321637406	1.060252561
Annual family income_13	0.517680642	0.445573995	1.16182867	0.245305066	−0.355628341	1.390989625
Annual family income_14	0.498880971	0.28148193	1.77233747	0.076338555	−0.052813474	1.050575416
Annual family income_15	0.473312539	0.26657928	1.775503854	0.07581473	−0.049173249	0.995798327

LR = logistic regression.

**Table 8:** Combined LR analysis: biomarkers and demographics.

Variable	Coefficient	Standard error	*z*	*P* > *z*	[0.025	0.975]
Constant	2.245443788	1.084002696	2.071437457	0.038317933	0.120798504	4.370089072
Alanine aminotransferase (ALT) (U/L)	0.019034869	0.004137902	4.600125969	4.22236E−06	0.010924582	0.027145156
Albumin, refrigerated serum (g/L)	−0.078554066	0.016652119	−4.717361622	2.38923E−06	−0.111192219	−0.045915913
Bicarbonate (mmol/L)	−0.057440042	0.018546301	−3.097115898	0.001954135	−0.093790791	−0.021089293
Glucose, refrigerated serum (mmol/L)	0.138788432	0.036546365	3.797598817	0.000146105	0.067157557	0.210419307
Iron, refrigerated serum (µmol/L)	−0.043716845	0.00740374	−5.904697807	3.53294E−09	−0.058228174	−0.029205515
Phosphorus (mmol/L)	−0.68695118	0.284582524	−2.413890954	0.015783185	−1.244732927	−0.129169433
Total protein (g/L)	−0.002269245	0.012360597	−0.183587003	0.85433746	−0.026496015	0.021957525
Triglycerides, refrigerated serum (mmol/L)	0.50003518	0.066828871	7.482322737	7.30527E−14	0.369050593	0.631019767
Uric acid (µmol/L)	0.007219509	0.000666623	10.8299754	0	0.005912928	0.008526089
Gender_2	0.401549734	0.108296798	3.707863391	0.000209015	0.189288009	0.613811459
Race/Hispanic origin_2	−0.058321084	0.22355183	−0.26088395	0.794182003	−0.496482672	0.379840503
Race/Hispanic origin_3	−0.704648029	0.167715448	−4.20144976	2.65211E−05	−1.033370308	−0.375925751
Race/Hispanic origin_4	−0.383353226	0.182649438	−2.098847009	0.035830389	−0.741346123	−0.025360328
Race/Hispanic origin_5	−1.59142183	0.177674602	−8.956946056	0	−1.93966405	−1.243179609
Education level—adults 20+_2	−0.142025876	0.227279212	−0.624896026	0.5320393	−0.587493132	0.303441379
Education level—adults 20+_3	0.104835286	0.213000207	0.492183964	0.6225893	−0.31264512	0.522315693
Education level—adults 20+_4	0.386304172	0.211157441	1.829460378	0.06733067	−0.027564413	0.800172757
Education level—adults 20+_5	0.248483318	0.221145764	1.123617803	0.2611752	−0.18496238	0.681929016
Marital status_2	−0.092817615	0.182363122	−0.50897141	0.610772264	−0.450249334	0.264614104
Marital status_3	−0.091527051	0.156290173	−0.585622561	0.558129184	−0.397855789	0.214801687
Marital status_4	−0.195359154	0.247365081	−0.789760436	0.429667689	−0.680194714	0.289476405
Marital status_5	−0.224218175	0.12670931	−1.769547752	0.076802509	−0.472568422	0.024132073
Marital status_6	0.070588832	0.170915797	0.413003555	0.679604009	−0.264406131	0.405583794
Annual family income_2	0.308342521	0.344758181	0.894373324	0.37112218	−0.367383515	0.984068556
Annual family income_3	−0.047601981	0.293459875	−0.162209506	0.871140875	−0.622783337	0.527579374
Annual family income_4	0.016471004	0.284348081	0.057925496	0.953807974	−0.540851234	0.573793242
Annual family income_5	0.367490003	0.290787767	1.263773942	0.206311177	−0.202454021	0.937434027
Annual family income_6	0.304696465	0.268711812	1.13391541	0.256830032	−0.221978687	0.831371617
Annual family income_7	0.571768136	0.276918457	2.064752715	0.038946404	0.02900796	1.114528312
Annual family income_8	0.349879274	0.289963692	1.206631326	0.227574157	−0.218449562	0.918208111
Annual family income_9	0.471046665	0.302576436	1.556785687	0.119521377	−0.12200315	1.06409648
Annual family income_10	0.588340991	0.319488099	1.841511447	0.065546645	−0.037855682	1.214537665
Annual family income_12	0.128150311	0.352529428	0.363516634	0.716218989	−0.562807368	0.819107991
Annual family income_13	0.400776839	0.445573995	0.899461916	0.368406672	−0.472548191	1.27410187
Annual family income_14	0.374659889	0.28148193	1.331026432	0.183180314	−0.177044694	0.926364472
Annual family income_15	0.342622771	0.26657928	1.28525657	0.198702641	−0.179872618	0.86511816

LR = logistic regression.

Both methodologies consistently highlighted bicarbonate and female gender as robust predictors, validating their biological relevance. The hybrid approach in Table 8 captured broader interactions (e.g., glucose, race) through ML-enhanced prioritization, while Table 7 emphasized strict statistical inference. This dual perspective underscores the value of combining traditional hypothesis testing with data-driven feature analysis to comprehensively interpret variable impacts.

By leveraging FI, SHAP, and LIME analyses, we gained comprehensive insights into the critical features driving our ML models, allowing for more informed interpretation and application of the models in practical scenarios.

## 4. Discussion

Obesity remains a persistent public health challenge globally, particularly in developed nations.^[43]^ The condition predisposes individuals to various metabolic complications including diabetes mellitus,^[44]^ hypertension,^[45]^ and respiratory disorders.^[46]^ While ML has gained prominence in medical research, existing studies predominantly focus on lifestyle factors and conventional biochemical markers (e.g., blood glucose,^[47]^ plasma lipids^[48]^) when investigating obesity-related parameters. Although emerging research has explored alternative predictors such as gut microbiota^[17]^ and general population characteristics,^[18]^ methodological limitations persist. Notably, a preliminary Spearman correlation analysis between hematological indices and BMI was reported in a small-scale study (n = 476),^[19]^ highlighting the need for larger validation cohorts.

Previous ML applications in this domain demonstrate variable success: Liu et al^[17]^ developed an Support Vector Machine model (classification accuracy: 0.716) using fecal metagenomic data from 2262 Chinese individuals; Chen et al^[19]^ implemented an extreme learning machine to distinguish overweight subjects (n = 225) from healthy controls (n = 251) based on serum biochemical profiles; Lin et al^[18]^ constructed predictive models using anthropometric features (primarily based on waist circumference, hip perimeter, female sex, and systolic blood pressure) from 5236 participants (2011–2012), though without external validation.

Our investigation addresses these limitations through comprehensive utilization of the NHANES dataset combined with rigorous internal–external validation protocols, ensuring robust model generalizability. Leveraging a cohort exceeding prior studies in scale (including external validation subsets), we systematically characterize novel obesity–biomarker relationships—focusing on electrolyte dynamics and hepatic/renal function indices—with BRF and LR models demonstrating critical methodological superiority. These algorithms achieved cross-validation accuracy stability (≤1.9%: BRF: 0.700→0.719; LR: 0.706→0.718), minimal AUC degradation in external testing (≤2.4%: BRF: 0.789→0.765; LR: 0.803→0.806), and resilient performance under data perturbations (≤1.1% accuracy variation with noise/outliers). This integrative approach advances mechanistic insights into metabolic dysregulation through clinically translatable computational frameworks.

In this study, we used ML methods to establish models of BMI and common social factors and biochemical markers for the 2017–2018 population, using the 2015–2016 population as external validation. We divided the BMI scores into 2 groups: <25 and ≥ 25, and attempted to distinguish between them. After constructing 6 ML models, we found that our models have significant potential in distinguishing between < 25 and ≥ 25 BMI. The differences between external validation and the primary model were minimal, and most internal validation scores were within the 95% confidence interval of bootstrap internal validation and bootstrap + OOB internal validation. However, we cannot ignore that ANN and AdaBoost may have overfitting issues during model construction, and XGBoost performance deteriorated in external validation. The BRF and LR models performed well in terms of scores, internal validation, external validation, and robustness, demonstrating strong generalizability and practical applicability.

The reasons for the various outcomes of these models can be explained. AdaBoost, as an ensemble learning method, improves overall model performance by combining multiple weak classifiers and gradually adjusting their weights to focus more on misclassified samples from previous rounds. This adjustment process may make the model increasingly complex, leading to overfitting. ANN’s overfitting is due to its high complexity, with numerous parameters and layers providing strong fitting capabilities, potentially overfitting the training data and failing to generalize well to unseen data. Although XGBoost did not exhibit overfitting, its generalizability decreased in external validation, similar to ANN. This could be due to differences in laboratory sampling methods between the 2 groups of data. For example, in 2015–2016, LDH was measured using the Beckman Coulter UniCel DxC 800/660i system (U/L; Beckman Coulter, Inc., Brea), while in 2017–2018, the Roche Cobas 6000 system was used (IU/L; Roche Diagnostics, Basel, Switzerland), leading to discrepancies in experimental methods and results. This is why we did not unify the data from different years for modeling. Despite this, we still established BRF and LR models that performed well in all aspects.

Besides triglycerides and glucose, which have been proven to be directly related to obesity,^[49]^ our study highlighted the significant contribution of phosphorus to the model and statistical analysis. Phosphorus was a crucial indicator of obesity in both LR analysis and statistical analysis. Although previous studies have shown that both high and low phosphorus levels may lead to obesity,^[50,51]^ the impact of phosphorus intake on obesity remains unclear.^[52]^ Studies indicate that higher consumption of phosphorus-added foods is associated with higher BMI, with women being more affected than men due to differences in bioavailability.^[52]^ Our results show a significant negative correlation between phosphorus levels and high BMI in both statistical and LR analyses. This means that higher blood phosphorus levels (within the NHANES measurement range) are associated with a more normal weight, which differs from the conclusions of the above studies. This discrepancy may be due to the former focusing on phosphorus intake, while our study directly examines blood phosphorus concentration, providing a more direct insight.

Uric acid is another key finding in our study, with significant importance in LR and statistical analyses as well as other models where it had high FI, SHAP, and LIME contributions. Liu et al^[53]^ found a positive correlation between urinary uric acid excretion levels and obesity/abdominal obesity in type 2 diabetes patients without chronic kidney disease. Wei et al^[54]^ found an association between albumin and hyperuricemia, with higher serum albumin in hyperuricemia patients, supporting our XGBoost findings. Primo et al^[55]^ directly confirmed the role of uric acid in obesity.

Furthermore, bicarbonate, ALT, and ALP demonstrated significant roles in both our modeling and statistical analyses. Prior research^[56]^ has established an association between lower bicarbonate levels and elevated BMI, a finding corroborated in our statistical analyses and LR results. This observation contrasts with the hypercapnia observed in obesity hypoventilation syndrome, typically linked to BMI ≥ 30 kg/m² and daytime hypercapnia (PaCO₂ ≥45 mm Hg).^[57]^ The discrepancy may stem from differences in BMI thresholds: our study defined the outcome at BMI = 25, suggesting that within the BMI 25 to 30 range, the respiratory-driven impact on bicarbonate homeostasis may gradually outweigh contributions from other organs—likely renal mechanisms. Recent hepatic function studies reinforce these findings. Ramesh et al^[58]^ conducted a cohort analysis of 1028 voluntary blood donors, demonstrating a positive association between BMI and ALT activity. Our results align with the growing consensus that obesity-related metabolic markers predominantly reflect hepatic and renal dysfunction. The well-documented association between obesity and nonalcoholic fatty liver disease^[59]^ further supports this paradigm. Genetic evidence from Kjaergaard et al^[60]^ identified pleiotropic links between high BMI and impaired renal function, consistent with emerging data on obesity as an independent risk factor for chronic kidney disease.^[61]^ Notably, Yau et al^[62]^ proposed a bidirectional interaction between renal function and adiposity. Our findings collectively reinforce the intricate interdependence between BMI and hepatorenal homeostasis.

In our study, gender, race, and income also played significant roles in model interpretation, especially in LR, with “other race—including multiracial” being the highest. Heymsfield et al^[63]^ used the NHANES database to explain different BMI values across races, finding that skeletal morphology and genetic mechanisms regulate fat and skeletal muscle mass differently across populations. Anekwe et al^[64]^ suggested that socioeconomic factors contribute to individual and community obesity, viewing it as a form of socioeconomic disadvantage. They also mentioned that metabolic syndrome is significantly higher in Black and Hispanic Americans, with female obesity rates inversely correlated with educational levels.

One of the main strengths of this study is the large sample size, which enhances the robustness of the analysis. Additionally, the use of both internal and external validation provides strong evidence for the model’s reliability. The minimal change in ACC after accounting for confounding factors further demonstrates the stability of the model. The NHANES database is widely recognized as an essential global resource, especially for epidemiological research, disease prevention, and health policy development.

However, despite the use of internal and external validation, the external validation data (2015–2016) were still derived from the same data source (NHANES), which may overestimate the generalizability of the model. Consequently, the predictive performance of the model may differ when applied to populations from other countries or different demographic groups.

A potential source of bias is the difference in detection methods between the 2 NHANES datasets (2015–2016 and 2017–2018), such as differences in instruments and reagents used. These discrepancies may introduce systematic errors, particularly when modeling across different time periods, possibly leading to data drift. To mitigate this issue, we avoided merging the 2 datasets for model development. Instead, we used the 2017–2018 dataset for model training and internal validation, while the 2015–2016 dataset was used for external validation. Although this approach reduced the overall sample size to some extent, it ensured the rationality and robustness of the model.

Another limitation is the inclusion of self-reported data (e.g., diet and physical activity), which may be subject to recall bias or social desirability bias, potentially affecting model predictive performance. However, by utilizing GA for feature selection and incorporating ML models with strong noise resistance (such as XGBoost and BRF), we were able to alleviate some of these biases to a certain extent.

For the interpretability of the ANN model, we applied the FI method. However, due to technical constraints, we were unable to use more advanced interpretation methods like LIME and SHAP for detailed explanations. In future work, model interpretability could be further improved by leveraging high-performance computing platforms.

Finally, the inclusion of demographic and income-related features in the model may introduce noise, which could lead to overfitting, especially in models like ANN and AdaBoost. Fortunately, except for these 2 models, no signs of overfitting were observed in the other models.

Despite these limitations, we successfully established ML models based on common biochemical indicators from the NHANES database. The models demonstrated strong performance, providing a valuable tool for risk prediction and clinical decision support.

## 5. Conclusion

In summary, we developed 6 ML models utilizing the NHANES database, with both internal and external validation confirming the reliability and practicality of these models. Our investigation highlights the impact of renal function parameters (including uric acid, bicarbonate, BUN, creatinine, and osmolality) and socioeconomic factors on BMI, particularly emphasizing the previously understudied association between serum phosphorus levels and BMI. Future directions should focus on: elucidating the molecular mechanisms underlying phosphorus-mediated adiposity regulation through prospective cohort studies, with particular attention to its interplay with calcium-phosphate homeostasis and endocrine modulation; developing multidimensional risk assessment tools integrating renal function with social determinants of health (e.g., neighborhood poverty index, educational attainment), prioritizing validation of BRF and LR models in primary care settings; conducting interventional clinical trials to evaluate dietary phosphorus restriction strategies for obesity management, with special consideration of ethnic variations in phosphorus metabolism. We recommend incorporating hepatic/renal function biomarkers into public health policymaking and establishing an integrated prevention framework that links metabolic health monitoring with community resource allocation.

## Acknowledgments

We, the research team, acknowledge the essential contributions of our authors: XC, BZ, HJ, HL, CX, HL, YC, and HC who generously contributed to this study. Their cooperation and involvement were essential for the successful completion of our research. Their cooperation and involvement were essential for the successful completion of our research.

The data utilized in this study were obtained from the National Health and Nutrition Examination Survey (NHANES) database, which is maintained by the NCHS. NHANES is a crucial nationwide survey. We acknowledge the NHANES program for providing access to the database, which significantly facilitated the progress of our work.

Special thanks to Xiaoli Chen, the corresponding author, for her insightful guidance and constructive feedback throughout the research process. Her expertise and encouragement have been invaluable.

Finally, we extend our appreciation to all individuals who directly or indirectly contributed to this project but are not mentioned here. Their support and encouragement are deeply appreciated.

## Author contributions

**Methodology:** Xiaoli Chen, Bingyu Zhu, Haiyang Jiang.

**Project administration:** Xiaoli Chen.

**Resources:** Xiaoli Chen, Bingyu Zhu, Haiyang Jiang, Haiyan Lan, Chunyu Xie, Hongmei Lin, Yonglingzi Chen, Hong Chen.

**Software:** Xiaoli Chen, Bingyu Zhu, Haiyang Jiang, Haiyan Lan, Chunyu Xie, Hongmei Lin, Yonglingzi Chen, Hong Chen.

**Writing – original draft:** Xiaoli Chen, Bingyu Zhu, Haiyang Jiang.

**Writing – review & editing:** Xiaoli Chen, Bingyu Zhu, Haiyang Jiang.

**Supervision:** Haiyang Jiang.

## Supplementary Material



## References

[1] Bessell E Markovic TP Fuller NR. How to provide a structured clinical assessment of a patient with overweight or obesity. Diabetes Obes Metab. 2021;23(Suppl 1):36–49. 33621413 10.1111/dom.14230

[2] Fisher M. Foreword: evaluation and management of overweight and obesity in children and adolescents. Curr Probl Pediatr Adolesc Health Care. 2020;50:100872. 32952064 10.1016/j.cppeds.2020.100872 PMC7499052

[3] Weir CB Jan A. BMI classification percentile and cut off points. In: StatPearls. StatPearls Publishing; 2024. 31082114

[4] Curtin LR Mohadjer LK Dohrmann SM. National health and nutrition examination survey: sample design, 2007-2010. Vital Health Stat 2. 2013:1–23. 25090039

[5] Johnson CL Dohrmann SM Burt VL Mohadjer LK. National health and nutrition examination survey: sample design, 2011-2014. Vital Health Stat 2. 2014:1–33. 25569458

[6] Fryar CD Kruszon-Moran D Gu Q Ogden CL. Mean body weight, height, waist circumference, and body mass index among adults: United States, 1999-2000 through 2015-2016. Natl Health Stat Report. 2018:1–16. 30707668

[7] Schetz M De Jong A Deane AM. Obesity in the critically ill: a narrative review. Intensive Care Med. 2019;45:757–69. 30888440 10.1007/s00134-019-05594-1

[8] Safaei M Sundararajan EA Driss M Boulila W Shapi’i A. A systematic literature review on obesity: understanding the causes & consequences of obesity and reviewing various machine learning approaches used to predict obesity. Comput Biol Med. 2021;136:104754. 34426171 10.1016/j.compbiomed.2021.104754

[9] Delpino FM Dos Santos Rodrigues AP Petarli GB. Overweight, obesity and risk of multimorbidity: a systematic review and meta-analysis of longitudinal studies. Obes Rev. 2023;24:e13562. 36929143 10.1111/obr.13562

[10] Dikaiou P Björck L Adiels M. Obesity, overweight and risk for cardiovascular disease and mortality in young women. Eur J Prev Cardiol. 2021;28:1351–9. 34647583 10.1177/2047487320908983

[11] Han H Li R Fu D. Correlation between bone density, bone metabolism markers with lipid metabolism markers and body mass index. BMC Musculoskelet Disord. 2024;25:162. 38378530 10.1186/s12891-024-07284-6 PMC10877819

[12] Varda NM Medved M Ojstersek L. The associations between some biological markers, obesity, and cardiovascular risk in Slovenian children and adolescents. BMC Pediatr. 2020;20:81. 32085704 10.1186/s12887-020-1978-5 PMC7033855

[13] Choi RY Coyner AS Kalpathy-Cramer J Chiang MF Campbell JP. Introduction to machine learning, neural networks, and deep learning. Transl Vis Sci Technol. 2020;9:14. 10.1167/tvst.9.2.14 PMC7347027 32704420

[14] Shah M Naik N Somani BK Hameed BMZ. Artificial intelligence (AI) in urology-current use and future directions: an iTRUE study. Turk J Urol. 2020;46(Supp. 1):S27–39. 32479253 10.5152/tud.2020.20117 PMC7731952

[15] DeGregory KW Kuiper P DeSilvio T. A review of machine learning in obesity. Obes Rev. 2018;19:668–85. 29426065 10.1111/obr.12667 PMC8176949

[16] Colmenarejo G. Machine learning models to predict childhood and adolescent obesity: a review. Nutrients. 2020;12:2466. 32824342 10.3390/nu12082466 PMC7469049

[17] Liu W Fang X Zhou Y Dou L Dou T. Machine learning-based investigation of the relationship between gut microbiome and obesity status. Microbes Infect. 2022;24:104892. 34678464 10.1016/j.micinf.2021.104892

[18] Lin W Shi S Huang H Wen J Chen G. Predicting risk of obesity in overweight adults using interpretable machine learning algorithms. Front Endocrinol (Lausanne). 2023;14:1292167. 38047114 10.3389/fendo.2023.1292167 PMC10693451

[19] Chen H Yang B Liu D. Using blood indexes to predict overweight statuses: an extreme learning machine-based approach. PLoS One. 2015;10:e0143003. 26600199 10.1371/journal.pone.0143003 PMC4658146

[20] Chen TC Clark J Riddles MK Mohadjer LK Fakhouri THI. National Health and nutrition examination survey, 2015-2018: sample design and estimation procedures. Vital Health Stat 2. 2020:1–35. 33663649

[21] Gnip P Vokorokos L Drotár P. Selective oversampling approach for strongly imbalanced data. PeerJ Comput Sci. 2021;7:e604. 10.7717/peerj-cs.604 PMC8237317 34239981

[22] Wang J. Prediction of postoperative recovery in patients with acoustic neuroma using machine learning and SMOTE-ENN techniques. Math Biosci Eng. 2022;19:10407–23. 36032000 10.3934/mbe.2022487

[23] Yang F Wang K Sun L Zhai M Song J Wang H. A hybrid sampling algorithm combining synthetic minority over-sampling technique and edited nearest neighbor for missed abortion diagnosis. BMC Med Inform Decis Mak. 2022;22:344. 36581862 10.1186/s12911-022-02075-2 PMC9801640

[24] Forrest S. Genetic algorithms: principles of natural selection applied to computation. Science. 1993;261:872–8. 8346439 10.1126/science.8346439

[25] Schober P Vetter TR. Logistic regression in medical research. Anesth Analg. 2021;132:365–6. 33449558 10.1213/ANE.0000000000005247 PMC7785709

[26] Zhang Z. Decision tree modeling using R. Ann Transl Med. 2016;4(15):275. 27570769 10.21037/atm.2016.05.14 PMC4980381

[27] Che D Liu Q Rasheed K Tao X. Decision tree and ensemble learning algorithms with their applications in bioinformatics. Adv Exp Med Biol. 2011;696:191–9. 21431559 10.1007/978-1-4419-7046-6_19

[28] Rigatti SJ. Random forest. J Insur Med. 2017;47:31–9. 28836909 10.17849/insm-47-01-31-39.1

[29] Anaissi A Kennedy PJ Goyal M Catchpoole DR. A balanced iterative random forest for gene selection from microarray data. BMC Bioinf. 2013;14:261. 10.1186/1471-2105-14-261 PMC3766035 23981907

[30] Tianqi C Carlos G. XGBoost: a scalable tree boosting system. CoRR abs/1603.02754. arXiv preprint arXiv. 2016

[31] Li K Zhou G Zhai J Li F Shao M. Improved PSO_AdaBoost ensemble algorithm for imbalanced data. Sensors (Basel). 2019;19:1476. 30917599 10.3390/s19061476 PMC6471212

[32] Geubbelmans M Rousseau A-J Burzykowski T Valkenborg D. Artificial neural networks and deep learning. Am J Orthod Dentofacial Orthop. 2024;165:248–51. 38302219 10.1016/j.ajodo.2023.11.003

[33] Bayman EO Dexter F. Multicollinearity in logistic regression models. Anesth Analg. 2021;133:362–5. 34257197 10.1213/ANE.0000000000005593

[34] Habibzadeh F. Data distribution: normal or abnormal? J Korean Med Sci. 2024;39:e35. 38258367 10.3346/jkms.2024.39.e35 PMC10803211

[35] Horn JL Engstrom R. Cattell’s scree test in relation to Bartlett’s chi-square test and other observations on the number of factors problem. Multivariate Behav Res. 1979;14:283–300. 26821851 10.1207/s15327906mbr1403_1

[36] Hazra A Gogtay N. Biostatistics Series Module 3: comparing groups: numerical variables. Indian J Dermatol. 2016;61:251–60. 27293244 10.4103/0019-5154.182416 PMC4885176

[37] West RM. Best practice in statistics: use the Welch t-test when testing the difference between two groups. Ann Clin Biochem. 2021;58:267–9. 33562996 10.1177/0004563221992088

[38] Li H Johnson T. Wilcoxon’s signed-rank statistic: what null hypothesis and why it matters. Pharm Stat. 2014;13:281–5. 24943680 10.1002/pst.1628

[39] Pandis N. The chi-square test. Am J Orthod Dentofacial Orthop. 2016;150:898–9. 27871717 10.1016/j.ajodo.2016.08.009

[40] Nahm FS. Receiver operating characteristic curve: overview and practical use for clinicians. Korean J Anesthesiol. 2022;75:25–36. 35124947 10.4097/kja.21209 PMC8831439

[41] Musolf AM Holzinger ER Malley JD Bailey-Wilson JE. What makes a good prediction? Feature importance and beginning to open the black box of machine learning in genetics. Hum Genet. 2022;141:1515–28. 34862561 10.1007/s00439-021-02402-z PMC9360120

[42] Gramegna A Giudici P. SHAP and LIME: an evaluation of discriminative power in credit risk. Front Artif Intell. 2021;4:752558. 34604738 10.3389/frai.2021.752558 PMC8484963

[43] Zukiewicz-Sobczak W Wróblewska P Zwoliński J. Obesity and poverty paradox in developed countries. Ann Agric Environ Med. 2014;21:590–4. 25292135 10.5604/12321966.1120608

[44] Mehran L Mousapour P Khalili D. BMI variability and incident diabetes mellitus, Tehran Lipid and Glucose Study (TLGS). Sci Rep. 2022;12:18370. 36319811 10.1038/s41598-022-22817-6 PMC9626493

[45] Peng N Kuang M Peng Y. Associations between TyG-BMI and normal-high blood pressure values and hypertension: cross-sectional evidence from a non-diabetic population. Front Cardiovasc Med. 2023;10:1129112. 37168658 10.3389/fcvm.2023.1129112 PMC10164981

[46] Yang W Yang Y Guo Y Guo J Ma M Han B. Obesity and risk for respiratory diseases: a Mendelian randomization study. Front Endocrinol (Lausanne). 2023;14:1197730. 37711902 10.3389/fendo.2023.1197730 PMC10497775

[47] Lin Z Feng W Liu Y. Machine learning to identify metabolic subtypes of obesity: a multi-center study. Front Endocrinol (Lausanne). 2021;12:713592. 34335479 10.3389/fendo.2021.713592 PMC8317220

[48] Gerl MJ Klose C Surma MA. Machine learning of human plasma lipidomes for obesity estimation in a large population cohort. PLoS Biol. 2019;17:e3000443. 31626640 10.1371/journal.pbio.3000443 PMC6799887

[49] Al Akl NS Haoudi EN Bensmail H Arredouani A. The triglyceride glucose-waist-to-height ratio outperforms obesity and other triglyceride-related parameters in detecting prediabetes in normal-weight Qatari adults: a cross-sectional study. Front Public Health. 2023;11:1086771. 37089491 10.3389/fpubh.2023.1086771 PMC10117653

[50] Anderson JJ. Potential health concerns of dietary phosphorus: cancer, obesity, and hypertension. Ann N Y Acad Sci. 2013;1301:1–8. 23848306 10.1111/nyas.12208

[51] Obeid OA. Low phosphorus status might contribute to the onset of obesity. Obes Rev. 2013;14:659–64. 23679666 10.1111/obr.12039

[52] Duong CN Akinlawon OJ Noel SE Tucker KL. Association between dietary intake of phosphorus and measures of obesity in the Jackson Heart Study. J Nutr. 2024;154:2188–96. 38795746 10.1016/j.tjnut.2024.05.014 PMC11282490

[53] Liu F Chen S Zhao W. Urine uric acid excretion levels are positively associated with obesity and abdominal obesity in type 2 diabetes patients without chronic kidney disease. Diabetes Metab Syndr Obes. 2021;14:4691–703. 34880638 10.2147/DMSO.S335558 PMC8646115

[54] Wei C Li T Xuan X Hu H Xiao X Li J. Serum albumin predicts hyperuricemia in patients with idiopathic membranous nephropathy. Clin Nephrol. 2021;96:191–8. 34142949 10.5414/CN110524

[55] Primo D Izaola O de Luis D. Resistin/uric acid index as a marker of metabolic syndrome in females with obesity. Int J Obes (Lond). 2023;47:393–8. 36864118 10.1038/s41366-023-01287-4

[56] Lambert DC Abramowitz MK. Obesity and the risk of low bicarbonate: a cohort study. Kidney Med. 2021;3:498–506.e1. 34401717 10.1016/j.xkme.2021.02.006 PMC8350812

[57] Shah NM Shrimanker S Kaltsakas G. Defining obesity hypoventilation syndrome. Breathe (Sheff). 2021;17:210089. 35035556 10.1183/20734735.0089-2021 PMC8753617

[58] Ramesh V Saraswat S Choudhury N Gupta RK. Relationship of serum alanine aminotransferase (ALT) to body mass index (BMI) in blood donors: the need to correct ALT for BMI in blood donor screening. Transfus Med. 1995;5:273–4. 8646290 10.1111/j.1365-3148.1995.tb00213.x

[59] Polyzos SA Kountouras J Mantzoros CS. Obesity and nonalcoholic fatty liver disease: from pathophysiology to therapeutics. Metabolism. 2019;92:82–97. 30502373 10.1016/j.metabol.2018.11.014

[60] Kjaergaard AD Teumer A Witte DR. Obesity and kidney function: a two-sample Mendelian randomization study. Clin Chem. 2022;68:461–72. 34922334 10.1093/clinchem/hvab249 PMC7614591

[61] Sikorska D Grzymislawska M Roszak M Gulbicka P Korybalska K Witowski J. Simple obesity and renal function. J Physiol Pharmacol. 2017;68:175–80. 28614766

[62] Yau K Kuah R Cherney DZI Lam TKT. Obesity and the kidney: mechanistic links and therapeutic advances. Nat Rev Endocrinol. 2024;20:321–35. 38351406 10.1038/s41574-024-00951-7

[63] Heymsfield SB Peterson CM Thomas DM Heo M Schuna JM. Why are there race/ethnic differences in adult body mass index-adiposity relationships? A quantitative critical review. Obes Rev. 2016;17:262–75. 26663309 10.1111/obr.12358 PMC4968570

[64] Anekwe CV Jarrell AR Townsend MJ Gaudier GI Hiserodt JM Stanford FC. Socioeconomics of obesity. Curr Obes Rep. 2020;9:272–9. 32627133 10.1007/s13679-020-00398-7 PMC7484407

