# Supplementary material for: A comprehensive analysis of BMI prediction using machine learning and biochemical markers: Insights from NHANES data
Source: Medicine (Baltimore). 2025 Jul 11;104(28):e42781. doi: 10.1097/MD.0000000000042781 (PMC12263063; doi:10.1097/MD.0000000000042781)
Supplement: Supplementary file 1 [file medi-104-e42781-s001.pdf]

Table(S1)The parameters of LR were adjusted

	solver	penalty	max_iter
LogisticRegression	lbfgs	L2	10000

Table(S2)The parameters of DT and RF were adjusted

	n_estimators	max_depth	min_samples_leaf	min_samples_split	max_features	criterion	Class_weight
DecisionTreer	-	3	4	2	6	gini	balanced
RandomForest	50	3	1	2	9	gini	balanced_subsample
	oob_score						
DecisionTreer	-						
RandomForest	true						

Table(S3)The parameters of XGboost were adjusted

	n_estimators	max_depth	min_child_weight	nthread	scale_pos_weight	gamma	colsample_bytree	subsample	reg_alpha	reg_lambda	learning_rate
XGboost	22	2	7	5	0.3	0.35	0.43	0.6	0.6	2	0.3

Table(S4)The parameters of Adaboost were adjusted

	n_estimators	learning_rate
Adaboost	28	0.5

Table(S5)The parameters of base_estimator parameters

	max_depth	min_samples_leaf	min_samples_split	max_features	criterion	Class_weight
base_estimator	2	12	15	6	gini	balanced

Table(S6)The parameters of ANN were adjusted

	Dense	activation	kernel_initializer	input_shape	Dropout
Neural Network	64	relu	GlorotUniform()	(input_dim,))	0.1
	Dense	activation	kernel_initializer	kernel_regularizer	Dropout
	64	relu	GlorotUniform()	L2	0.1
	Dense	activation			
	1	sigmoid			
	learning_rate	loss	metrics	monitor	
	0.001	binary_crossentropy	accuracy	val_loss	
	patience	restore_best_weights	class_weight	epochs	batch_size
	10	true	{0: 4.0, 1: 1.0}	100	64
	verbose	validation_split			
	1	0.2			
